# A cross-cultural concept analysis of healing in nursing: a hybrid model

**DOI:** 10.1186/s12912-023-01404-8

**Published:** 2023-08-01

**Authors:** AkramSadat SadatHoseini, Habib Shareinia, Shahzad Pashaeypoor, MohammadMehdi Mohammadi

**Affiliations:** 1 Department of Pediatric Nursing, School of Nursing and Midwifery, Tehran University of Medical Sciences, Tehran, Iran; 2 Department of Medical-Surgical Nursing and Basic Sciences, School of Nursing & Midwifery, PhD Candidate of Nursing, Tehran University of Medical Sciences, Tehran, Iran; 3 Department of Community Health and Geriatric Nursing, School of Nursing & Midwifery, Tehran University of Medical Sciences, Tehran, Iran; 4 School of Nursing and Midwifery, Kermanshah University of Medical Sciences, Kermanshah, Iran

**Keywords:** Concept analysis, Healing, Nursing, Culture

## Abstract

**Background:**

As a familiar yet abstract and vague concept for nurses, healing is affected by the cultural needs of different communities. The concept of healing is nowadays recommended in nursing theories, and its clarification can develop healing-based nursing care. The present study was conducted to objectify and clarify the concept of healing in nursing care.

**Methods:**

The present research employed a hybrid concept analysis model developed by Schwartz-Barcott and Kim. The conceptual analysis model of Walker & Avant was used in the theoretical phase, i.e., literature review, where relevant articles in PubMed, ISI, Google Scholar, Scopus, SID and Magiran were reviewed irrespective of publication time. Unstructured interviews were conducted with ten participants in the field data collection phase. A final analysis was performed by combining the two phases.

**Results:**

The theoretical phase identified healing characteristics such as balancing and hope-making originated from mental and spiritual states. Analyzing the data in the fieldwork stage extracted five main themes, i.e. “comprehensive psychophysical health”, “cure, a small part of healing”, “healing, a spiritual recovery”, “an individual’s own role in healing” and “healing, an unexpected event”. During the patient care process, nurses can help patients heal by establishing appropriate communication and comprehensive understanding of the patients by designing and implementing appropriate interventions and integrating healing strategies into their comprehensive care measures.

**Conclusions:**

The concept of healing in nursing care is a general and complex process, and different people can interpret it differently on their road to health. Properly understanding the concept of healing enables nurses to assist patients in achieving health and healing through proper communication, holistic care, empowering patients to perform self-care and providing spiritual care.

## Background

Healing is a familiar word in the healthcare delivery system; nevertheless, its culture-dependent nature has prevented features of healing from being elucidated. Healthcare workers therefore require obtaining a clear picture of healing and its applications. Despite continuous use of healing concept in nursing and the related theories, it is differently defined in different cultures and communities [1, 2]. Developing the original concept of healing and specifying all dimensions therefore require complete understanding of the mentioned concept. A concept can be better understood and then applied and even measured in the next steps in case its dimensions are identified.

Nurses help patients achieve health and healing [3]. Many theorists made suggestions; e.g. Watson defined the aim of nursing as helping with achieving a higher degree of coordination of mind, body and spirit by establishing different processes of self-awareness, self-respect, self-healing and self-care. Watson believed that both patients and caregivers are involved in self-healing [4].

The importance of healing turned caring-healing modalities into a core concept in Watson’s theory, based on which holistic nurses who are aware of caregiving use caring-healing modalities such as music, therapeutic touch, aromatherapy and relaxation to promote recovery and help maintain a proper relationship. According to Watson’s theory, nurses nowadays use therapeutic touch as a healing intervention to relieve pain and anxiety and accelerate wound healing, improve physical symptoms, reduce the side effects of chemotherapy and normalize blood pressure [5].

Healing is strongly influenced by culture and religion and affects the patients’ decisions about their type of treatment and even the continuation or discontinuation of treatment and care [6, 7]. Rassool found the care models proposed in the west not to be well applicable to Islamic countries, and dominant care concepts in a nursing model to be sometimes totally incompatible with the concepts of the Islamic culture such as healing and spiritual dimensions of care [8]. Addressing patients’ culture, including their spiritual and religious beliefs is one of the requirements of holistic care because religious beliefs about health and disease affect patients’ care decisions [9]. The concept of patient healing has therefore been debated for long in different cultures, and the same debate can be applied to nursing [10]. Therefore, nurses’ understanding of the healing concept in different cultural spectra can help evaluate the reasons for patients’ decisions and choices, and their approach to changes during the illness [6, 7].

According to Islamic principles, nurses should lay the groundwork for healing in patients through comprehensively empowering them, and realize the potential for healing present in all humans through providing sympathetic care [11]. Islam places great emphasis on the concept of healing. In the holy Quran, Verse 82 of Surah Al-Isra [17:82] (And we send down of the Qur’an that which is healing and mercy for the believers, but it does not increase the wrongdoers except in loss), known as the healing verse, reflects this belief in Islamic texts. Healing in the Islamic culture is therefore regarded as an endogenous rather than marginal or exogenous dimension of patient recovery, and everybody seeks healing as soon as they get sick, and the nurse’s job is to help patients achieve it [11].

As a concept in treatment and care plans that is influenced by culture, healing should be defined based on the Iranian culture as well. Given the inadequate understanding of the concept of healing, its vague application in nursing, and nurses’ general unwillingness to design healing-based care plans due to the lack of rational evidence for it [12], elucidating and analyzing this concept can help promote its clinical application in patients and improve the existing nursing theories.

The hybrid model of conceptualization developed by Schwartz-Barcott and Kim is appropriate for clarifying concepts such as healing that are influenced by cultural factors and conventional sciences and definitions, and the final definition shall also include these cultural factors [13, 14].

Considering the different worldviews of the Islamic Iranian culture on health, disease and death, the concept of healing needs to be defined within this culture as a widely-used concept that is influenced by culture in treatment and care programs. By gaining awareness about this concept, nurses can help patients recognize the internal and external forces affecting their health. The present study was therefore conducted in Iran to clarify the concept of healing in nursing and take account of the effect of sociocultural which is influenced by religious context and background on this concept using the hybrid model [15].

## Methods

### Design

Given the varying perspectives and opinions on healing and its diverse definitions in different cultures, religions and beliefs, clarifying this concept can help improve its application in nursing. The hybrid model developed by Schwartz-Barcott and Kim was used to analyze the concept of healing because it is suitable for use in the intended context and since it can clarify ambiguous concepts in clinical practice. The hybrid model is a method for the conceptualization and evolution of a concept in its context. This model consists of theoretical, fieldwork and final analysis phases [16].

**Theoretical phase**: The purpose of this stage was to develop an appropriate context for an in-depth analysis and redefinition in the subsequent stages [17]. We used the concept analysis method of Walker and Avant (2005) with the following eight steps: (1) Selecting a concept; (2) Determining the aims or purposes of analysis; (3) Identifying all uses of the concept; (4) Determining the defining attributes; (5) Constructing a model case; (6) Constructing borderline, related, contrary, and invented cases; (7) Identifying antecedents and consequences; (8) Defining empirical referents [17]. Data were analyzed using Elo and Kyngas’ content analysis [18]. Therefore, the following steps were taken to examine the phrases that provided meanings, definitions, and attributes for the concept through the careful study of each source:

1. Preparation: Reading the whole text to gain an overall understanding of the concept, immersion in the data, determining the unit of analysis.
2. Organization: Defining primary codes, creating subcategories and categories.
3. Reporting: Reporting results and analysis process through categorization and storyline.

The concept and dimensions of healing were thoroughly investigated at the end of the search, and a theoretical definition of healing and its dimensions was ultimately obtained.

#### Fieldwork

At this stage, the concept formed in the theoretical phase was reinforced and refined by performing qualitative interviews [17]. In the present study, fieldwork was necessary for two reasons. First, there were not enough texts about the concept of healing in Iranian culture to help the researcher achieve sufficient conceptualization. Second, according to Islamic-Iranian culture, it was necessary to interview and obtain the opinions of some experts who had closely experienced and observed the phenomenon of healing to enrich the data. The present study fieldwork was performed using a qualitative approach in Tehran, Iran in 2020. Purposive sampling was employed to select subjects with adequate knowledge and experience in the study context. The inclusion criteria comprised speaking Persian, speech and hearing health and willingness to be interviewed, knowledge of the concept, and experience of communication with patients who had experienced the concept of healing. The snowball sampling method was used to find key informants; based on the information about healing retrieved from the search, the researcher determined with whom to conduct the first interviews. Then, the interviewees introduced some other key informants. According to the study objectives, Unstructured face-to-face interviews were conducted with 10 experts, including 4 nurses, 2 Physician (internist), 2 Muslim clergyman and 2 General public (A housewife and a PhD student).

Since healing is a multidimensional concept and the views of clinical staff, clerics and the general public can be different, groups of people with different views were interviewed. Nurses and head nurses from internal and pediatric wards with long work experience with chronically ill and critically ill patients or experience in the emergency room, and a physician who had experience in this field and was interested in the subject were among the primary participants. Some participating nurses and physicians had several experiences of patients’ healing by appealing to God and the Shia Imams, and then they introduced clerics or members of the general public who had relevant information and experience. To this end, unstructured interviews were conducted individually wherever was convenient for the participants, such as a hospital or an office. The researcher obtained permission to record the interviews with an audio recorder and take notes during the interviews, if necessary. The interviewees were informed of their right to leave the study at any time and to take all relevant documents from the researcher. All the participants were asked about two key questions, i.e. “What does healing mean to you?” and “What crosses your mind as you hear the word of healing?”. Further questions raised as per the participants’ responses to explore the concept dimensions included “Please provide an example of this”, “What do you mean by this?” and “Please elaborate on this issue”. Each interview lasted 30–40 min depending on the personal preferences and conditions of the participants. All the research ethical issues were observed in the interviews. The data were analyzed in the MAXQDA10 software using the content analysis proposed by Graneheim and Lundman [19]. To this end, the following steps were taken: (1) Transcribing the entire interview immediately after each interview; (2) Reading the whole text for a general understanding of its content, (3) Determining semantic units and basic codes, (4) Categorizing similar primary codes into more comprehensive categories, and (5) Determining the main categories.

Data saturation was reached after eight interviews; however, two more interviews were also conducted with nurses to enrich the work.

#### Final analysis

This stage involved combining the theoretical analysis with the insights obtained from the empirical observations and reported findings [17]. The definitions and concepts obtained from the two previous stages were merged into a comprehensive definition encompassing all the features.

At this stage, the codes extracted from the theoretical stages were compared with the codes extracted from the field work. After the analysis, a comprehensive definition of healing in nursing care was achieved. At this stage, the findings of the field work were prioritized over the theoretical findings based on participants’ experience and the Islamic culture of the studied society.

### Credibility and robustness of data

Guba and Lincoln’s four criteria were used to ensure the value of the data and the reliability of the results [26]. The researcher had prolonged engagement with the participants and the research topic, which helped gain the trust of the participants and understand the studied setting. Member checks were also used to confirm the accuracy of the data and codes; for this purpose, after coding, the transcripts were given to the participants to ensure the accuracy of the codes and interpretations. The researchers tried to select participants with maximum variation in terms of age, occupation, and level of education. The transcripts and extracted codes and categories were reviewed and approved by several associate researchers.

## Results

### The first phase: the theoretical phase

1. **Selecting a concept**.

Given the use of concepts such as healing and self-healing in Islamic-Iranian culture and their frequent use by patients in the process of treatment and care, especially in hard-to-treat diseases, and that according to researchers, this concept is still ambiguous in nursing, we decided to clarify and define this concept for use in the patient care process.

2. **Determining the aims or purposes of analysis**.

This analysis aimed to clarify and summarize the existing definitions of healing in books and articles as well as the perspective of specialists and patients, and finally to provide a final definition of healing in nursing care.

3. **Identifying all uses of the concept**.

Different texts introduce different definitions and applications for healing, some of which in dictionaries in general and in Islamic sources are as follows:

Merriam-Webster Dictionary defines healing as “to make free from injury or disease”, “to make well again” and “to cause (an undesirable condition) to be overcome” [20]. According to Cambridge Dictionary, “A bad or terrible emotional condition either ends or improves after healing” [21]. Oxford Dictionary defines healing as “to become healthy again” and “to make something healthy again” [22]. Dehkhoda Dictionary defines healing as “health and recovery from an illness”(23). Mo’in Encyclopedic Dictionary defines healing as “becoming well after an illness, recovery, health and cure” [24]. Amid Dictionary defines healing as “making well, free someone from a disease, making healthy and recovery from an illness” [25]. The Quran defines healing as salvation from material and spiritual decline and collapse, which involves both spiritual and physical treatment. Being healed by the Quran is equivalent to salvation from all evils, which is achieved in the absence of fear with hopes for a bright future and a gentle spirit and results in decent and reasonable conduct that brings psychophysical health. The Quran called itself healing rather than medicine given that medicine may temporarily treat a disease, whereas healing is a perfect treatment and health that eradicates the disease [26]. Absolute recovery and health literally defined as healing is therefore emphasized in all aspects in Islam.

4. **Determining the defining attributes**.

The concept of healing in nursing was first selected and a comprehensive non-systematic review of literature was accordingly performed in databases such as PubMed, ISI, Google Scholar, Scopus, SID and Magiran using keywords such as healing, concept analysis and nursing and their Persian equivalents. The eligible articles included fully-accessible original studies and review articles in English and Persian related to the study concept with no limitations on their publication date. Seventeen out of 37 initially-extracted articles were selected for analysis based on the inclusion criteria (10 articles in English and 7 in Persian).

Table 1 presents the results of searching through the databases in the theoretical phase by the studies conducted outside Iran and Table 2 presents the results associated with the studies conducted in Iran.

**Table 1:** A summary of certain features of the healing concept in the studies conducted outside Iran

Analysis and result	Method	Title	Author, Reference, Country
Healing is a human experience toward optimal coordination, balance and interaction that depends on different spiritual, physical, mental and social dimensions. Healing can be holistically affected by age, genetics, experiences, the environment and relationships.	Concept analysis	Holistic health care: Evolutionary conceptual analysis	Ziebarth (27), United States
As a comprehensive restoration and recovery process of the body, mind, and spirit, healing causes a movement toward positive changes and full self-actualization in the presence or absence of diseases.	Concept analysis	Healing, a concept analysis	Firth and Bellanti (1), United States
Healing is an unpredictable subjective process that involves a complete transformation, spiritual transcendence and a reinterpretation of life. Nurses can play a role in the healing process through transpersonal care and patients through their beliefs and inherent therapeutic abilities and natural aid. Analyzing the concept of healing in nursing can help clarify the responsibility and role of nurses in this process.	Concept analysis	Healing: the journey from concept to nursing practice	McElligott (28), United States
Healing is a transformative process that changes individuals in anticipated and unanticipated manners and produces a new entity. Individuals achieve excellence under suffering and pains and change from old to new. This is a positive transformation in both the healer and those to be healed.	A book review article	Florence nightingale today healing, leadership, global action	Dossey BM (29), United States
According to Watson’s theory, nursing aims at helping with achieving a higher degree of coordination of mind, body and spirit by establishing different processes of self-awareness, self-respect, self-healing and self-care. Emphasis is placed on self-conscious changes while focusing on the relationship between healing and care and changing toward spiritualizing health versus limited medical vision. Moving in this path and delivering professional nursing care requires new standards, guidelines and models to be developed.	A review article	The attending nurse caring model: integrating theory, evidence and advanced caring–healing therapeutics for transforming professional practice	J. Watson and F. Roxie (30), United States
Healing refers to hoping for relieving pains and meeting an easy death; healing means putting the patient in a more normal and facilitated situation for a happy life; healing is accompanied with changes in values; what used to be important to the patient will no longer matter, as personal values evolve after healing. Healing means being freed from annoying values. Healing helps the patient as a whole be liberated from annoying situations and values and move toward no pain, no agony, and peace. What used to be important to the patients and caused mental conflict will no longer be valued after healing and values undergo dramatic changes.	A qualitative study	The meaning of healing near the end of life	Gauthier (31), United States
Explaining the difference between cure and healing; cure means improving physical and emotional symptoms or putting a stop to or slowing down the destructive process of a disease; medicinal and surgical interventions are performed in curing. Healing refers to gradual awareness of a more profound sense of self, body, mind and spirit; healing is a concept with a holistic origin. Healing focuses on the individual and cure on the disease. Healing emphasizes human integrity, puts human at the center of attention during care and it is associated with the realization of human’s inner energy in terms of physical, mental and spiritual dimensions.	A review study	Healing the spirit	McGlone ME (32), Philippines
Healing is a natural process with an internal origin, which appears to be linked with human nature and to aim at restoring the individual’s balance that is inherent in human nature. Healing is natural rather than magical or mystical; healing is an internal process that occurs to restore balance. Healing comprises complex internal processes and occurs in the absence or despite external interventions.	A review article	On healing, wholeness and the haelan effect	Quinn JF (33), United States
From an Islamic point of view, the concept of care is considered a spiritual umbrella that covers the basic needs of the patient according to the Holy Quran and the hadiths of the Prophet (PBUH). Extensive and in-depth research should be performed on asking healing of the Quran as a holy book. Investigating and being inspired by healing approaches in the Quran, Muslim and non-Muslim researchers are recommended to design and implement modern nursing care models. Three different approaches to healing and health improvement in the holy Quran include the legal approach such as balance in nutrition, abstinence from alcohol and tobacco, regular exercise, prayer and fasting, the guidance approach involving the introduction of general rules of daily living and the direct approach, i.e. the effect of the holy Quran itself on different body systems.	A review article	The crescent and Islam: healing, nursing and the spiritual dimension. Some considerations towards an understanding of the Islamic perspectives on caring	GH Rassool (8), United Kingdom
For these patients, the meaning of healing differed from physical treatment and encompassed physical, social, spiritual, and mental dimensions. Acceptance of the illness, submission to it, hope, freedom from suffering, overcoming the illness, and positive feelings were among the meanings of healing. Most of the patients associated healing with belief in a higher power.	A qualitative study	The Meaning of Healing to Adult Patients with Advanced Cancer	Namisango et al. (34), Uganda

The results of the above studies in non-Iranian cultures suggest that healing is a step-by-step process that uses natural forces within an individual to lead the person to general recovery, self-actualization, painlessness, and peace in all dimensions of health, including physical, mental, psychological and spiritual. This harmony resulting from healing causes balance in all physical and non-physical dimensions. Dynamic and active participation of the individual, having interest and motivation, self-control, and self-care activities play an important role in this route. Using their healing science and art, cultural and spiritual care, and holistic support of patients, nurses play a constructive role in empowering and helping patients during the healing process, and can be effective in designing new models in this field.

**Table 2:** A summary of certain features of the healing concept in studies conducted in Iran

Analysis and result	Method	Title	Author ,Reference
Nurses must use God’s healing power in patient healing. Nurses are in charge of empathizing, sympathizing, giving hope and being kind in their interactions with the patient. In these therapeutic interactions, not only does the patient achieve healing and health, but also the nurse attains the status of a benefactor, and both achieve excellence.	A review article	Nursing in Prophet Mohammad narration and life: Explanation of concept of nursing based on narration of Mohammad Prophet	Akram al-Sadat Sadat Hosseini et al. [11]
Treatment differs from healing. Treatment refers to receiving medications to remove a material cause, whereas healing is basically a psychological and spiritual process. The Quran is healing as it improves both body and soul diseases and strengthens the immune system for future confrontations.	Editorial	The Quran is an agent for healing and resistance against diseases	Mostafa Ghanei [35]
The main role of healing gardens is to provide their users with a completely clean and healthy environment and to comfort them. Being inspired by the wisdom of Avicenna, designers of the environment should pay a special attention to the role of healing landscapes in human psychophysical health and health promotion.	A review article	A comparative investigation of healing landscape criteria and wise Abu Sina’s recommendations in the Book of Law	Faezeh Tavakolian and Azadeh Shahcheraghi [36]
Seeking healing from the Quran should be seriously detailed. Believing in the Quran as a healer turns every verse into healing and a cure for all pains. Psychophysical diseases can be basically treated with medical procedures. Treatment is part of healing. The Quran is a perfect healer rather than a therapist.	A review article	Healing from the perspective of the Quran	Mahmoud Gholamreza Mirzaie [26]
One turns to their God as the only healer in most of the prayers recommended in Islam during diseases. Prayer is a healing process. Quran verses recommend praying to the unique Creator for healing and cure.	A review article	The pole of prayer in health promotion: a perspective on prophetic tradition and new scientific documentation	Jahangir et al. [37]
Iranian traditional medicine referred to a physician as a hakim, who combined prayers and cures for treatment. The long history of using prayers for treatment reminds us that prayer is a divine gift with a profound religious dimension. The instructions for prayer and healing in Iranian traditional medicine are based on four principles, i.e. praying by putting a hand on patient head, writing prayers, transferring prayers to water or foods and remotely praying	A review article	Interpretation of verse 82 of Surah Al-Isra concerning prayer and healing.	Jahangir and Maftun [38]
According to these patients, spiritual healing was affected by supernatural forces; to achieve spiritual healing, in addition to using approaches such as telepathy and energy therapy, they also sought help from God as the Supreme Power through prayer, so that they could reach a higher level of physical and mental health and continue living more energetically.	A hybrid method	Spiritual healing from Iranian cancer patients’ viewpoints: A hybrid concept analysis	Rafii et al [39]

Research on healing in Iran suggests that asking the Quran as a perfect healer for help, creating a lively environment, believing in God as the only healer, seeking health from God and getting help from Quranic prayers and Prophet Mohammad’s manners are examples of seeking healing for patients. Nurses can lay the foundations for patients’ healing in a holistic manner by empathizing with and being kind and giving hope to them. The Iranian culture defines healing as a comprehensive recovery process, although it emphasizes its spiritual dimension.

Findings of various studies showed that human beings do not spare any efforts to achieve health when they are sick and in pain. To restore their health, people seek help from health and medical staff, including physicians and nurses for their medical, surgical and care measures; furthermore, they try to achieve healing gradually via motivation with the help of others and using their inner forces and the spiritual forces they believe in. With holistic care, nurses can learn about patients’ culture and spiritual beliefs, and help them seek health and excellence through connecting to the main source of energy they believe so as to achieve a comprehensive balance and healing.

5. **Constructing a model case**.

The case was a 50-year-old woman with a spinal cord injury of about 8 years, complete bilateral paraplegia at the T7-T8 level due to a spinal artery problem, and bladder and bowel incontinence who used a wheelchair. After about 4 years of follow-up and visiting many physicians, she lost hope in treatment. After a while, she tried to gradually gain hope, asking God for healing and regaining health in her prayers. Amid frequent hospitalizations, she asked the physicians and nurses who had a better medical relationship with her if she could be free of this paralysis and disability by going to Mashhad and appealing to Imam Reza (one of the holy men in Shiite Islam). Among them were nurses who sympathetically listened to her and encouraged her to do so. The patient then went to Mashhad with the help and encouragement of her family after finding a positive inner feeling for her treatment through healing. After two days of staying near the tomb of the holy person and praying to God and asking the holy person to mediate before God for the treatment of her paralysis and disability, she suddenly heard a whisper at a night that said “everything is over, stand up now”. She woke up and stood up, but she could not walk yet. At that moment, she was filled with joy and enthusiasm, and it seemed that there was no pain, sorrow and grief in her body and soul. She felt that a force was activated in her and she was released from the disease forever. After some physiotherapy, the patient recovered completely and was able to walk without assistance. After this incident, medical records before and after healing were examined. Complete neurological examinations (MRI, NCV and EMG) after healing showed that the patient’s condition was almost normal, which was also approved and recorded in the Commission for Healed Affairs of Astan Quds Razavi (consisting of eminent clerics, expert physicians, judges and shrine clerks).

6. **Other cases**.

### A borderline case

The case was a 71-year-old man with diabetes and coronary artery disease admitted to the cardiac surgery ward after coronary angiography. According to the physician, he had to undergo coronary artery bypass surgery. Due to fear and anxiety about the surgery and its complications, he refused the surgery and was discharged from the ward against medical advice. After discharge, he prayed to God for the cure of his heart disease. After some days of prayer and appealing to God, he gradually felt better. His dyspnea decreased while calmness and positive feelings grew in him. In the follow-ups during this time, he told nurses that atherosclerosis in in his cardiac arteries were eliminated and that he was healed by God. He said that he was completely well and did not need surgery with the careful continuation of the medical treatments. However, his heart condition aggravated after a while and he was hospitalized for a while. He eventually died of a heart attack while sleeping at home. Despite his prayers, this patient unfortunately died because he failed to achieve inner strength to heal and gained a false idea of healing instead of pursuing treatment.

### Contrary case

The case was a 47-year-old man with advanced colon cancer under treatment, which unfortunately had failed. He was very upset and anxious because he saw death imminent. One of his relatives suggested that he be healed by strengthening his inner faith and asking God for healing. The patient became very angry and rebuked the person and considered such things superstitious and undocumented. He called strengthening the healing inner force by seeking help from a higher power ridiculous, unreal and fabricated by the illusions of a group of religious people. In this case, the person did not believe in strengthening his inner forces with the help of a higher power and denied it, and even considered those who use this power to be ignorant and superstitious.

7. **Identifying antecedents and consequences**.

#### Antecedents

They are events that must exist before the concept can be created [16]. The antecedents of the concept of healing include patients’ communication with themselves and others [1, 32, 40], the onset of reconstruction process in physical, mental and spiritual dimensions [2, 9, 33], the adequacy or inadequacy of medical interventions [41], and creation of a special and emotional relationship in the person [1].

**Consequences**: They include events and conditions that occur as a result of the concept [16]. In this analysis, healing yielded the following consequences: restoration, recovery and comprehensive balance in body, mind and soul [28, 30, 37], which have caused positive evolution and changes in the person, whether the disease has been completely eliminated or not. The healed person is aware of the changes in themselves [29, 30] and is deeply satisfied with achieving this condition.

8. **Defining empirical referents**.

Given that the concept of healing is really subjective and is individually experienced, Firth et al. suggest that a hybrid (quantitative and qualitative) method including interviewing patients to understand their experiences of healing to comprehend changes in emotions, changes in values and self-concept, and quantitative measurement of physiological changes can be useful in measuring the concept of healing [1]. Miller et al. also proposed a hybrid method to measure the concept of healing [42]. However, no tool was found to measure the concept of healing or the healing process.

The theoretical phase results revealed seven features or main categories for the concept of healing as follows (Table 3):

#### Comprehensive change

This category included the four subcategories of total recovery, full actualization, changes in all dimensions, and all-round peace and comfort, which indicated deep and comprehensive change in the person after healing. Patient-centered teamwork with a multidisciplinary and professional team is required for healing. Healing is complete physical, mental, and spiritual regeneration and transformation toward self-actualization and transcendence, which can result in a change in values [27, 28, 31, 32, 35, 36].

#### Balance

This category included two subcategories of coordination in different physical and non-physical dimensions and favorable balance which express an all-around balance. Experiencing healing, one moves toward coordination and balance in various physical, mental, psychological and spiritual dimensions, and achieves high degrees of balance in body, soul, and mind [27, 30, 34].

#### Process

The process of healing involves three dimensions of step by step towards the goal, gradual changes, and interconnected internal and external activities. Healing is a process that occurs dynamically and comprehensively over time, however, it occurs along with characteristics such as unpredictability, excitement and turmoil, and it ultimately leads to an outcome [1, 28, 29, 33].

#### Awareness

In healing, people are aware of the positive changes in their states and conditions and, while controlling their thoughts and actions, are engaged in this process actively and enthusiastically. This self-conscious change from within the person toward healing can also be considered a kind of spiritual change [8, 29, 30, 33].

#### Care

Nurses can play an effective role in the healing process by helping patients consciously transform and achieve comprehensive health and healing through comprehensive and loving interactions. Holistic care, patient empowerment, and cultural and spiritual care are the components of care in healing [11, 28, 30].

#### Quran as a healer

All-around healing of patients can occur by following the instructions and rules stated in the Qur’an, as well as through the direct impact of its verses on health [8, 26, 35].

#### Prayer

Praying during illness is a form of mental support for the patient [34, 37–39].

**Table 3:** Formation of categories and subcategories in the theoretical phase

Categories	Subcategories
Comprehensive change	Total recovery Full actualization Changes in all dimensions All-round peace and comfort
Balance	Coordination in different physical and non-physical dimensions Favorable balance
Process	Step by step toward the goal Gradual changes Interconnected internal and external activities
Awareness	Active participation Interest and motivation Having a sense of control
Care	Holistic support Patient empowerment Cultural and spiritual care
Quran as a healer	Health tips Lifestyle rules The effect of divine revelations
Prayer	Asking God for health Prayer as a supportive method

### The final definition of the theoretical phase

Healing is a multifaceted process and focuses on the whole human being. During pain and suffering, patients receive a kind of positive energy by establishing a deep relationship with themselves and therapists, and try to achieve health and balance in all physical and non-physical dimensions. In the worldview of Muslim patients regarding health, the concept of disease is associated with patience, prayer and hope. In Iranian Islamic culture, patients, in addition to benefiting from medical procedures, try to achieve health by following the Quranic instructions, seeking help from the divine verses of the Quran, asking the infinite divine power and appealing to the Prophet of Islam and his family, especially in hard-to-treat diseases. By recognizing this concept and understanding how this process is practiced, nurses can help patients accelerate regaining health. In other words, familiarizing patients and nurses with this concept can improve nurses’ professional performance.

### The second phase: fieldwork

Table 4 presents the demographic information of ten participants interviewed to complete the study.

**Table 4:** Demographic details of the participants in the fieldwork phase

Participant	Number/Gender	Mean age	Level of education
Nurse	2 women/2 men	38	3 undergraduates/1 postgraduate
Physician	2 men	36	2 internists
Muslim clergyman	2 men	45	1 undergraduate/1 postgraduate
General public	1 woman/ 1 man	33	1 undergraduate/1 Nursing PhD student

Data were coded based on the interviews, which yielded over 400 initial codes. Duplicate codes were eliminated, and the remaining codes were categorized by their differences and similarities. The findings of this stage were divided into five themes, each of which is briefly explained as follows:

### Comprehensive psychophysical health

The majority of the participants believed health has different dimensions, and therefore healing includes cure and health in all the psychophysical, mental and spiritual dimensions. Healing eliminates or reduces physical and mental injuries and problems. Healing encompasses different dimensions depending on the type of disease and patient conditions. A participant said, “Healing is the work performed by the health team, physicians, nurses, health workers, etc. as pharmaceutical, surgical, caregiving and supportive interventions to reduce patient problems” (P # 5).

Another participant said, “In acute diseases such as infectious diarrhea, the physical dimension of healing dominates its psychological aspect, whereas in chronic diseases, the mental and spiritual dimensions of healing prevail. The approach to the meaning and concept of healing differs according to the disease type…” (P # 6).

### Cure, a small part of healing

According to the participants, cure constitutes a small part of healing as a whole. Physicians and nurses are only tools and ways to achieve healing, which is something more than cure.

A participant added, “A physician is not the only way to achieve healing; for instance, completely treating a patient with incurable physical diseases such as a malignant tumors or chronic psychological problems by resorting to religious scholars in addition to using medical treatments also constitutes a kind of healing” (P # 1).

### Healing, a spiritual recovery

Healing caused by one’s progress in spiritual and religious affairs was the next theme obtained. One’s spiritual preparedness affects healing, as man naturally seeks an infinite power. This inspiration causes connection to a source.

A participant said, “The Quran itself is an example of healing. The soil of Karbala, where Imam Hussein, a righteous man in Islam, was martyred, is another example of healing by God. One’s beliefs are very important in these matters, as they may not work for everyone!” (P # 8).

### An individual’s own role in healing (self-healing)

According to the participants, the patients themselves play a key role in their healing. One’s motivation, will and hope are crucial in healing. Patients who love their life and desire to live are healed faster.

“… Healing does not occur unless you want it, help yourself and seek it wholeheartedly!” (P # 10).

### Healing, an unexpected event

The participants reported the sudden occurrence of healing in some cases. A participant said, “Healing is like a spark and the starting point of a dream. Even hearing about the healing of others affects our soul. When I realize that a patient has been healed, a feeling of joy comes over me” (P # 2).

The results of the interviews showed that when people are in a state of illness and disease, they voluntarily first seek therapeutic strategies, including medication and surgery, which are more used in curable diseases, and they consider these solutions part of healing. In Iranian Islamic culture and in the Shiite religion, the concept of healing is mostly spiritual, and it is believed that by praying to God and appealing to and asking the infallible Imams (holy people in Shiite culture), one can suddenly and permanently regain health even in hard-to-treat diseases.

### The third phase: final analysis

A more comprehensive and complete definition of healing was obtained at this stage compared to those of the two previous phases by merging their results using a perceptual and communicative analysis perspective. Putting together the conceptual features of healing derived from the theoretical and the field work phases showed that healing is a comprehensive and complex process with an excellent source in the Islamic culture and features such as balancing and hope giving originated from mental, spiritual, and religious states. The implicit or explicit antecedents of healing include establishing a special emotional relationship between the patients and their self, according to the culture and religion, others or a transcendental being such as God. The reconstruction and change process begin in all the physical, mental and spiritual dimensions of patients. The outcomes of healing include complete recovery of one’s body, mind and spirit and achieving comprehensive coordination and balance irrespective of whether or not the disease is completely treated. Fieldwork investigations suggested the participants identified healing as a general and great concept intertwined with religious and spiritual matters. They also found that care and treatment constitute part of healing, and that spirituality, religion and the patients themselves play an undeniable role in healing.

The present study found the healing process to differ from one patient to another. Given humans as unique creatures with different personal perceptions of phenomena and the fact that healing does not occur unless the patient seeks self-healing, the following definition can be proposed for the concept of healing in nursing care:

Healing is a complex process focused on human integrity during which one moves toward health, balance, and self-actualization with the help of treatments, care, potential internal healing forces and religious and spiritual sanctities associated with their culture and community. Healing is a process that differs from and goes beyond cure. Nurses can assist patients in achieving healing during the care process through properly communicating and acquiring a comprehensive understanding of them via designing and implementing appropriate interventions and integrating healing strategies into their comprehensive care measures.

#### Healing antecedents

The pain and suffering caused by the disease, the patient’s active role, effort and motivation, self-care, therapeutic and care measures, praying, seeking help from the Qur’an, appealing to the saints, establishing good communication with oneself, others, and the treatment team, establishing a special spiritual relationship with God, hope, nurses’ holistic care, and spiritual care.

#### Healing consequences

Physical and mental health, promoting self-care activities, empowering the patient on the road to health, self-actualization, freedom from suffering, vitality of the healthcare team, growth of the nursing profession, spiritual transformation, achieving spiritual health, a sense of happiness and satisfaction.

## Discussion

The present study was conducted to analyze the concept of healing in nursing care in an Iranian nursing context. As discussed in the review of literature, healing is a general and comprehensive concept with a focus on the body, mind and spirit for achieving comprehensive coordination and balance. Nurses can play a key role in assisting patients in achieving healing by establishing respectful and empathizing relationships with them. The complexity of the disease the patient is suffering and the risks of medical and surgical therapies appear effective in the patient’s understanding of this concept. Given the themes extracted in Materials and Methods, the participants emphasized the comprehensiveness of healing and the roles of spirituality and the patient in healing.

Firth and Bellanti found healing as a holistic concept to constitute a restoration and recovery process in the mind, body and spirit causing positive changes and helping find meanings, move toward self-actualization and eradicate the physical symptoms of the disease [1]. Gauthier states that healing frees the individual from suffering in general and changes their values [31]. Ziebarth [31] and McGlone ME [27] also considered healing as coordination and balance in different dimensions of health and emphasized that healing is a concept that focuses on an individual as a whole. In line with the present study, this finding suggests the comprehensiveness of healing in all human dimensions. Due to cultural and religious differences in the communities examined in our study, the process of achieving healing with the help of religious sanctities played a prominent role.

The hybrid method proposed by Miller et al. for measuring healing required considering its cultural differences [42]. Given the multidimensionality of the concept, social, cultural and spiritual contexts should be therefore addressed in the design of healing evaluation instruments.

Dossey introduced healing as a transformative process that changes patients in anticipated and unanticipated manners[29], whereas the present study identified healing as a predictable process with specific antecedents. According to Rassool, the holy Quran attributes the concepts of healing and health to balanced nutrition, exercise, doing religious practices, observing general instructions in life and the direct effect of the Quran on human body systems[8]. Despite being a predictable process, healing cannot be therefore predicted yet owing to the weakness of human knowledge.

According to Watson’s theory, nursing goals include the processes of self-awareness, self-respect, self-healing and self-care, and nurses can play a key role in healing by establishing appropriate therapeutic relationships with patients[30]. McElligott suggested that clearer dimensions of healing will help nurses better assist patients in the healing process [28]. Sadat Hosseini et al. mentioned that nurses should facilitate healing by comprehensively empowering patients [11] such that they can move on the path of healing with their divine nature, which is consistent with the present findings despite the differences between the examples in the religious thought.

Studies of Rassool [8], Ghanei [35], and Mirzaee [26] have suggested Quran as a means to reach healing, which is consistent with the findings of the present study considering Iran’s Islamic context. The Islamic culture believes following the divine commands of the Quran can provide health and the verses of the Quran are directly effective in treating patients.

Of the limitations of our study was the unsystematic review of the literature in the theoretical phase. Since the information of the people who were healed was confidential, we could not access them and interview them. Another limitation was the anonymity of these people because those who were actually healed were usually unwilling to be identified and interviewed, and usually those who thought they were healed or made false claims about it were willing to be interviewed by the research team. Another limitation was the language limitation of the texts that included only Persian and English texts. It is recommended that further studies be conducted with an in-depth search in other languages ​​to explain features of healing as much as possible. Given the fieldwork phase of this study was conducted at the outset of the COVID-19 pandemic, the concept that there may be a relationship between healing and COVID-19 was not yet formed in the minds of the research team. It is suggested that such a concept be considered in future studies.

## Conclusion

Healing is a general and complex concept that depends on culture and community. Through empathy, kindness and proper communication with patients, nurses can play a major role in helping them achieve healing while considering this concept an endogenous force in providing care. Nurses need to consider this concept an endogenous force in providing care to patients in order to motivate and support them in determining solutions by supporting their choices and thus helping them achieve peace and health. Applying the concept of healing in the clinic involves two important aspects in the nursing practice. First, despite personal belief or disbelief in healing, nurses should be aware that they can use patients’ inner healing power as one of the methods suggested to them. It is the patients’ right to be informed of it and, if desired, to be provided with the means to use healing through traveling to their holy places and furnishing patients and their families with conditions for prayer away from personal judgments or prejudices. Second, in addition to addressing the physical and mental dimensions of patients in the care process, nurses should be aware that according to some cultures such as Islam, disease is an opportunity for inner transcendence and strengthening of human beings, and healing is the external manifestation of this transcendence in the body and soul of patients. Nurses can help patients in this regard only if they are aware of the nature and process of healing. To clarify all the dimensions of the concept of healing, further studies are recommended to be conducted in other areas of health sciences or on patients of different age groups using diverse data collection methods. Given concepts as the building blocks of a theory, the present findings can contribute to developing nursing models and theories, assist in producing and developing tools to investigate this phenomenon, and provide more opportunities to apply the concept in clinical research and theoretical, educational and managerial areas of nursing.

## Data Availability

The datasets analyzed during the current study are not publicly available due confidentiality of the participants but are available from the corresponding author upon reasonable request.
